# Moderate and heavy metabolic stress interval training improve arterial stiffness and heart rate dynamics in humans

**DOI:** 10.1007/s00421-012-2486-6

**Published:** 2012-09-16

**Authors:** Mark Rakobowchuk, Emma Harris, Annabelle Taylor, Richard M. Cubbon, Karen M. Birch

**Affiliations:** 1 Health, Exercise and Active Life Research Unit, Sport and Exercise Sciences, Biological Sciences, University of Essex, Wivenhoe Park, Colchester, CO4 3SQ UK; 2 Multidisciplinary Cardiovascular Research Centre, University of Leeds, Leeds, UK

**Keywords:** Heart rate variability, Arterial compliance, Exercise training, Arterial stiffness, Heart rate modulation, Interval

## Abstract

Traditional continuous aerobic exercise training attenuates age-related increases of arterial stiffness, however, training studies have not determined whether metabolic stress impacts these favourable effects. Twenty untrained healthy participants (*n* = 11 heavy metabolic stress interval training, *n* = 9 moderate metabolic stress interval training) completed 6 weeks of moderate or heavy intensity interval training matched for total work and exercise duration. Carotid artery stiffness, blood pressure contour analysis, and linear and non-linear heart rate variability were assessed before and following training. Overall, carotid arterial stiffness was reduced (*p* < 0.01), but metabolic stress-specific alterations were not apparent. There was a trend for increased absolute high-frequency (HF) power (*p* = 0.10) whereas both absolute low-frequency (LF) power (*p* = 0.05) and overall power (*p* = 0.02) were increased to a similar degree following both training programmes. Non-linear heart rate dynamics such as detrended fluctuation analysis $({| {1 - \alpha_{1} }|})$ also improved (*p* > 0.05). This study demonstrates the effectiveness of interval training at improving arterial stiffness and autonomic function, however, the metabolic stress was not a mediator of this effect. In addition, these changes were also independent of improvements in aerobic capacity, which were only induced by training that involved a high metabolic stress.

## Introduction

Hypertension, as a major risk factor for cardiovascular and cerebrovascular disease is often preceded and predicted by arterial stiffness (Dernellis and Panaretou 2005). Arterial stiffness progresses throughout the lifespan contributing to greater arterial pressure and greater left ventricular myocardial oxygen consumption through higher afterload. Exercise training is an effective non-pharmacological intervention strategy shown to attenuate or reverse age-related arterial stiffening (Tanaka et al. 2000). Carotid artery stiffness is also involved in autonomic function and may be associated with sympatho-vagal irregularities (Rowe 1987). Thus, by attenuating progression or rectifying arterial stiffness, exercise training may also favourably impact sympatho-vagal balance (Monahan et al. 2001).

Effective exercise intensities and dosages that improve arterial stiffness have been described, although strength or resistance exercise training has demonstrated both negative (Miyachi et al. 2005) and equivocal adaptations (Rakobowchuk et al. 2005; Kawano et al. 2008). Recently, sprint interval training improved peripheral (popliteal) arterial stiffness (Rakobowchuk et al. 2008). Similarly, heart rate variability may be improved with endurance training suggesting an improvement in autonomic control (Seals and Chase 1989; Goldsmith et al. 1992; al-Ani et al. 1996), however, no study to date has examined the effects of interval exercise training upon autonomic cardiac modulations in young populations. Since interval training methods have been suggested to be more effective than traditional endurance exercise training in improving maximal aerobic capacity (Wisløff et al. 2007; Gibala and McGee 2008), and recent evidence from an observational longitudinal study emphasizes vigorous physical activity as a major factor modulating carotid artery stiffness (van de Laar et al. 2010), it may be inferred that a high metabolic stress is required for cardiovascular adaptation.

Muscle metabolic adaptation undoubtedly depends on the metabolic stress of each exercise session but favourable vascular adaptations may be independent of metabolic stress. Exercise domains related to lactate threshold (LT), critical power (CP), and $\dot{V}{\text{O}}_{2\max }$ have been defined to appropriately characterize metabolic stress (Rossiter 2011). However, studies that aimed to improve vascular stiffness with exercise training have rarely defined the exercise domain involved.

In the present study, we controlled factors involved in improving arterial stiffness and cardiac autonomic modulations with interval exercise training. We compared exercise-training programmes matched for duration and work but differing in overall metabolic stress by modulating duty cycles according to Turner et al. (2006). This resulted in one group experiencing a moderate (<LT) and the other group a heavy (>LT) metabolic stress. We hypothesised, contrary to longitudinal data, that heavy and moderate metabolic stress interval training would induce similar vascular and cardiac autonomic adaptations since the volume of training would be equal and overall shear similar. If supported, this would suggest metabolic stress is not a major modulator of these adaptations.

## Methods

### Participants

Healthy men and women (*n* = 7 men and 13 women) volunteered for the study (Table 1). Participants were free of risk factors associated with cardiovascular, pulmonary or metabolic disease, deemed safe to begin physical activity, and were not engaged in a regular training program. Other exclusion criteria included medication use, pregnancy and smoking. The experimental procedures and potential risks were explained prior to the study, and all participants provided written, informed consent. The local Ethics Committee at the University of Leeds approved the experimental protocol, which conformed to the Declaration of Helsinki.

**Table 1:** Subject characteristics at rest before (PRE) and after (POST) 6 weeks of either moderate or heavy intensity interval training

	Moderate (*n* = 9)		High (*n* = 11)		*p* value time
	PRE	POST	PRE	POST	
Age (years)	23.7 ± 3.4	–	23.1 ± 2.5	–	–
Height (cm)	173.9 ± 5.5	–	171.7 ± 12.8	–	–
Weight (kg)*	74.3 ± 12.2	73.2 ± 9.9	67.3 ± 13.6	67.0 ± 13.3	–
BMI (kg m^−2^)*	24.4 ± 2.2	24.3 ± 3.5	22.7 ± 3.1	22.6 ± 3.0	–
LV ejection duration (ms)	355 ± 20	356 ± 21	370 ± 32	382 ± 45	0.48
Brachial SBP (mmHg)^†^	112.0 ± 0	109.1 ± 7.0	112.0 ± 0	109.4 ± 6.9	0.01
Brachial MAP (mmHg)^†^	84.0 ± 0	83.0 ± 5.9	84.0 ± 0	82.0 ± 5.8	0.01
Brachial DBP (mmHg)^†^	70.6 ± 0	68.8 ± 5.8	70.6 ± 0	68.5 ± 5.7	0.03
Carotid PP (mmHg)	32.3	30.7	32.3	32.2	0.61
Peripheral PWV (m/s)	8.6 ± 1.3	8.9 ± 1.7	8.4 ± 1.3	8.4 ± 1.2	0.53
Carotid AIx (%)	−7.0 ± 14.9	−6.7 ± 10.5	−12.5 ± 18.0	−10.3 ± 15.0	0.63
Carotid wave reflection time (ms)	182 ± 37	188 ± 42	208 ± 29	217 ± 27	0.36
Carotid IMT (mm)	0.36 ± 0.07	0.39 ± 0.07	0.38 ± 0.08	0.39 ± 0.07	0.13

Data are mean ± SD

*BMI* body mass index, *IMT* intima-media thickness, *PWV* pulsewave velocity, *AIx* augmentation index

* Significant group difference (main effect for group *p* < 0.05). *p* values of time effects correspond to ANOVA when there were no differences at PRE and ANCOVA analysis when differences at PRE occurred (see “Results”)

^†^Adjusted means from the ANCOVA are presented

### Experimental protocol

Participants visited the laboratory for assessments prior to completing a 6-week exercise-training program (PRE). Maximal aerobic capacity ($\dot{V}{\text{O}}_{{ 2 {\text{peak}}}}$), resting ECG for heart rate variability, resting blood pressure and carotid artery diameters using ultrasound were determined. Participants were assigned to either a heavy exercise domain interval training (HEDIT) or a moderate exercise domain interval training (MEDIT) group in a matched fashion based on $\dot{V}{\text{O}}_{{ 2 {\text{peak}}}}$. The maximal work rate attained at $\dot{V}{\text{O}}_{{ 2 {\text{peak}}}}$ was used to determine the work rate of the intervals. All assessments were repeated following training at least 48 h after their last training session (POST). Female participants were tested ~2 week prior to the commencement of the training program to ensure that POST assessments were within the same phase of their individual menstrual cycle.

### Assessment of arterial stiffness

Visits to the laboratory were controlled for time of day. Participants were instructed to abstain from caffeine that morning and exercise for 24 h prior to PRE measurements. Participants were supine in a temperature-controlled (22–24 °C) room for 20 min prior to measures.

Carotid artery stiffness (β-stiffness index) and pressure wave morphology [augmentation index (AIx), wave reflection time] was assessed using a combination of Ultrasound imaging (Aspen, Acuson, Siemens Medical) and applanation tonometry (model SPT-301, Millar Instruments Inc., Texas, USA). These methods have been previously described (Rakobowchuk et al. 2008). Images were taken 2–3 cm proximal to the carotid artery bulb and this was ensured at follow-up through video feedback. Two video clips of 20 s were acquired at 15 Hz. Simultaneously a hand-held pressure transducer, sensitive to hold-down pressure, was held perpendicular to the contralateral carotid artery to acquire arterial blood pressure waveforms. Measurements of brachial blood pressure were also obtained for the purpose of calibrating the carotid waveforms (Omron M6, Milton Keynes, UK) as previously described (Rakobowchuk et al. 2008). The carotid waveform does not differ significantly from that obtained by means of invasive catheter (Kelly et al. 1989). It was assumed that DBP and MAP are similar in all conduit arteries in the supine position whereas SBP is amplified throughout the arterial tree. Mean and minimum BPs obtained from the carotid waveform were equated to the MAP and DBP of the brachial artery. The maximum of the BP waveform value recorded in the carotid artery was then used as an extrapolation point from the calibrated MAP and DBP.

All video clips used to determine artery stiffness were analysed by the same investigator using a semi-automated edge detection software program (Vascular Tools v.5, Medical Imaging Applications, Coralville, Iowa, USA). Subsequently, at least 20 measurements of diameter change were used to calculate β-stiffness index (O’Rourke et al. 2002) as:

1 $$ \beta{\text{-}}{\text{stiffness\;index}} = { \ln }\left( {{\text{SBP}}/{\text{DBP}}} \right)/\left[ {\left( {{\text{diameter}}_{ \max } - {\text{diameter}}_{ \min } } \right)/{\text{diameter}}_{ \min } } \right] $$

Peripheral upper-limb pulsewave velocity (PWV) was determined as an index of arterial stiffness using the same tonometer previously described from 20 to 40 waveforms obtained at the carotid and radial arteries. Pulse transit times were determined as the time delay between simultaneously acquired ECG R-waves, and the foot of the pressure wave (second derivative of this waveform). The distance the pulsewave travelled was measured as the difference between the distance from the sternal notch to the carotid and the sternal notch to the radial measurement site. PWV was calculated as the distance/time delay.

Carotid pressure waveform contours were analysed to obtain left ventricular ejection duration, carotid augmentation index, and reflection time of the blood pressure waveform. These were determined using previously described Matlab algorithms (Munir et al. 2008).

### Vascular structure measurements

Intima-media thickness (IMT) and minimum and maximal arterial diameters were determined from the carotid artery images. Mean arterial diameter was calculated as 1/3 × systolic diameter + 2/3 × diastolic diameter. IMT was determined from the average of twenty images acquired at end-diastole and each image involved between 150 and 200 diameters determined from the region of interest (Vascular Tools v.5, Medical Imaging Applications, Coralville, Iowa, USA).

### Assessment of heart rate variability

Ventricular depolarization was recorded by electrocardiograph (V5 configuration) using commercially available hardware (Powerlab model ML, ADInstruments, Colorado Springs, USA) and software (LabChart 7.03, ADInstruments, Colorado Springs, CO, USA) sampled at 20 MHz. Premature beats (i.e. >20 % shortening) were excluded manually and replaced with interpolated values and accounted for <1 % of each participants’ dataset. R–R intervals were interpolated at 4 Hz and detrending was performed using the smoothness priors method described by Tarvainen et al. (2002). The same duration (5 min) of data were analysed as established by the Taskforce (Malik et al. 1996).

### Linear heart rate variability

Analysis of HR variability was conducted with the aid of HRV Analysis Software Kubios 2.0 for Windows (The Biomedical Signal Analysis Group, Department of Applied Physics, University of Kuopio, Kuopio, Finland). The power spectral densities (PSD) were quantified from Welch’s periodogram and by 16th order AR modelling. Total, very low-frequency (VLF 0.0–0.04 Hz), low-frequency (LF 0.04–0.15 Hz), and high-frequency (HF 0.15–0.4 Hz) power was determined and compared before (PRE) and after (POST) training in both groups. In addition, normalized units (nu) of LF and HF were determined and the LF/HF ratio was calculated (Montano et al. 1994). The spectral parameters of LF and HF are often associated with physiological alterations of sympathetic and parasympathetic input to the heart. The spectral power within the LF portion is often described as a function of both sympathetic and parasympathetic input while the HF portion is widely accepted to originate from parasympathetic modulation of the heart rate (Berntson et al. 1997).

### Non-linear heart rate dynamics

Several non-linear components of HR variability were calculated, including detrended fluctuation analysis (DFA), which has been described previously elsewhere (Peng et al. 1995; Goldberger et al. 2000). Simply, DFA is a modified root mean-square analysis of a random walk (Peng et al. 1995). Details about the calculation of DFA are available (Peng et al. 1995; Goldberger et al. 2000). Values approaching α = 1.0 are considered the ideal balance between the predictable signal of Brownian noise (α = 1.5) and the complete unpredictability of white noise (α = 0.5) (Peng et al. 1995; Goldberger et al. 2000). In this study, we used the short-term (4–16 beats) scaling exponent (α_1_) based on previous research (Makikallio et al. 1997). Also since values that get closer to 1.0 indicate improvements, a difference score was calculated ($| { 1.0 - \, \alpha_{ 1} } |$) (Millar et al. 2009).

Sample entropy (SampEn), a measure of non-linear HRV was also determined. Values approaching 0 are considered highly regular and larger values represent greater complexity (Goldberger et al. 2000). Input variables for pattern recognition, *m* and *r* were set at 2 and 0.20 similar to previous studies (Costa et al. 2002).

Poincaré plots were constructed from the R–R interval data for each participant (Carrasco et al. 2001). These plots display each R–R interval as a function of the subsequent R–R interval in that time-series. The dimensions of this graphical representation describing the width and breadth of this relationship are determined (SD_1_ and SD_2_). SD_1_ describes the short-term variability and is often related to respiratory sinus arrhythmia while SD_2_ relates to long-term variability. We are most concerned with SD_1_ since the time-series used for the analysis is not long enough in duration to ensure adequate confidence in SD_2_.

### Time-domain heart rate dynamics

Several time-domain analyses were determined as recommended by the Task Force (Malik et al. 1996). The standard deviation of the N–N (SDNN: an estimate of overall HRV), the HRV triangular index (estimate of overall HRV), and the root mean square of the standard deviation of the R–R interval (RMSSD: an estimate of short-term HRV) were determined.

### $\dot{V}{\text{O}}_{{ 2 {\text{peak}}}}$ assessment

Participants performed a progressive exercise test [increasing 1 W every 4 s (females) or 1 W every 3 s (males)] on a cycle ergometer (Lode BV, Excalibur Sport V2.0, the Netherlands) to determine $\dot{V}{\text{O}}_{{ 2 {\text{peak}}}}$ and estimated lactate threshold (_e_LT) using an on-line gas collection system (Medgraphics D-Series, Medgraphics, Medical Graphics Corporation, St Paul, MN, USA). $\dot{V}{\text{O}}_{{ 2 {\text{peak}}}}$ was calculated as the maximal value over a 30 s period and was confirmed by a respiratory exchange ratio (RER) value >1.15, attainment of >95 % of age-predicted maximal heart rate and a rate of perceived exertion (RPE) >18 (1–20 scale). _e_LT was determined using standard non-invasive pulmonary gas exchange parameters (Beaver et al. 1986) and as utilized by Turner et al. (2006) when determining subsequent exercise domains.

### Training protocol

Participants completed 6 weeks of training, attending three supervised sessions per week and all sessions were attended (i.e. 100 % adherence). MEDIT and HEDIT training programs were based on previous work by Turner et al. (2006) involving alterations of duty cycles. Participants completed 30 (week 1–2), 35 (week 3–4), and 40 (week 5–6) min of exercise at each session preceded by a 2-min warm-up at 20 W. All exercise-training sessions were completed using a Lode Excalibur enabling virtually instantaneous changes in work rate. The MEDIT group completed repeated duty cycles of 10 s:20 s. These duty cycles consisted of 10 s of work completed at 120 % of their pre-training maximal work rate and 20 s of recovery at 20 W. This pattern repeated until the target duration was met. The HEDIT group completed duty cycles of 30 s:60 s in this same manner. By design, the protocols involved an identical total training volume and time commitment but differed regarding metabolic stress. Short duration duty cycles (e.g. 10 s work and 20 s recovery, 1:2 duty cycle) have been shown to have a lower metabolic stress than longer duration duty cycles (e.g. 30 s work and 60 s recovery; 1:2 duty cycle) despite the power-output, training duration and total work being similar between the two (Turner et al. 2006). Accordingly, if the work intervals (for 10 s) were at 120 % of the work rate achieved at $\dot{V}{\text{O}}_{{ 2 {\text{peak}}}}$ and recovery was at a work rate of 10–20 W (for 20 s) a metabolic stress below LT was achieved. Alternatively, longer duty cycles of 90 s (30 s at 120 % work rate at $\dot{V}{\text{O}}_{{ 2 {\text{peak}}}}$: 60 s at 10–20 W) cause a metabolic stress above LT but below critical power (CP). Consequently, the exercise-training programmes based on duty cycle alterations enable the modulation of metabolic stress within recognized exercise domains and the evaluation of this factor on vascular parameters.

### Statistical analysis

Normal distribution of data were assessed (Kolmogorov–Smirnov) and log transformed if non-normal. PRE data were examined for group differences via independent Student *t* test. Data were analysed using two-way mixed model ANOVA with group (HEDIT vs. MEDIT) and time (PRE vs. POST) factors. For variables with group differences at PRE, ANCOVA was used with PRE values held constant as covariates. Alpha was accepted as *p* ≤ 0.05. Linear regression analysis was performed to examine the relationship between initial arterial β-stiffness index and training-induced changes in β-stiffness index of the carotid artery. All values are presented as mean ± SD. Analyses were performed using SPSS (Version 18.0, IBM Corporation, Somers, NY, USA).

## Results

### Participants

**Fig. 1:**
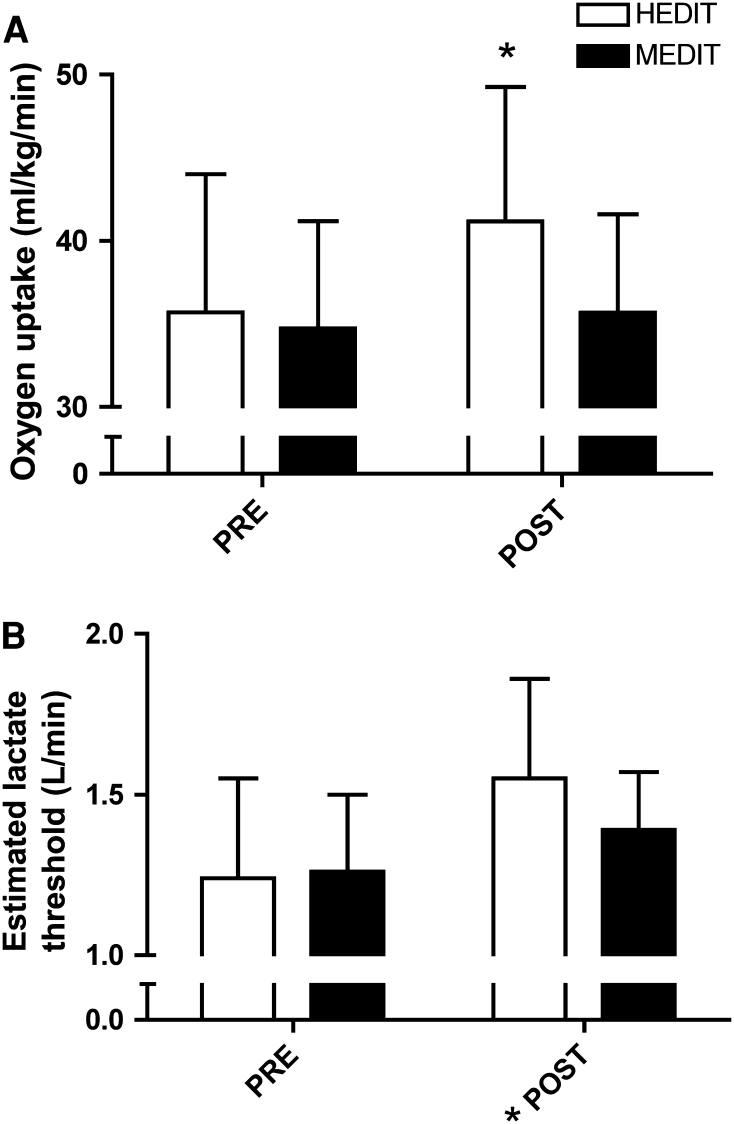
Maximal aerobic capacity and estimated lactate threshold. **a** $\dot{V}{\text{O}}_{{ 2 {\text{peak}}}}$ was improved in the HEDIT-trained group after 6 weeks of training, those in the MEDIT group did not change (interaction *p* = 0.05). **b** The estimated lactate threshold improved irrespective of training group (main effect for time, *p* < 0.04). *Significant change with training

Participant descriptors are in Table 1. Although matched according to $\dot{V}{\text{O}}_{{ 2 {\text{peak}}}}$ some PRE group differences in mass, BMI, and resting blood pressure existed, but all values fell within the healthy range. Absolute and relative $\dot{V}{\text{O}}_{{ 2 {\text{peak}}}}$ increased with training in the HEDIT group but were unaltered in the MEDIT group (Interaction: absolute *p* = 0.04, relative *p* = 0.05, Fig. 1a). _e_LT increased in both groups (time effect, *p* < 0.01; interaction, *p* = 0.11, Fig. 1b).

### Heart rate and arterial blood pressure

Heart rate reduced with training (HEDIT: PRE 65 ± 9 vs. POST 64 ± 10 bpm; MEDIT: PRE 71 ± 10 vs. POST 66 ± 11 bpm, time effect *p* = 0.03, interaction *p* = 0.21) but the magnitude of this reduction was not likely physiologically meaningful. Left ventricular ejection duration was unaltered in either group (time *p* = 0.48, interaction *p* = 0.55, Table 1). There were group differences at PRE in brachial arterial systolic (HEDIT PRE: 105.0 ± 9.2 mmHg vs. MEDIT PRE: 118.8 ± 15.9 mmHg, *p* = 0.03), diastolic (HEDIT PRE: 67.0 ± 6.0 mmHg vs. MEDIT PRE: 74.8 ± 7.6 mmHg, *p* = 0.02) and mean (HEDIT PRE: 79.7 ± 6.4 mmHg vs. MEDIT PRE: 89.1 ± 9.0 mmHg, *p* = 0.01) arterial pressures. After adjustment for these differences ANCOVA analyses revealed significant training-induced reductions (time effect SBP: *p* < 0.01, DBP: *p* < 0.01, MAP: *p* < 0.01, Table 1), but no interactions. Carotid pulse pressure was not different at PRE between groups (*p* = 0.58) and no training effects were noted (time effect, *p* = 0.61, Table 1).

### Central arterial structure and stiffness

**Fig. 2:**
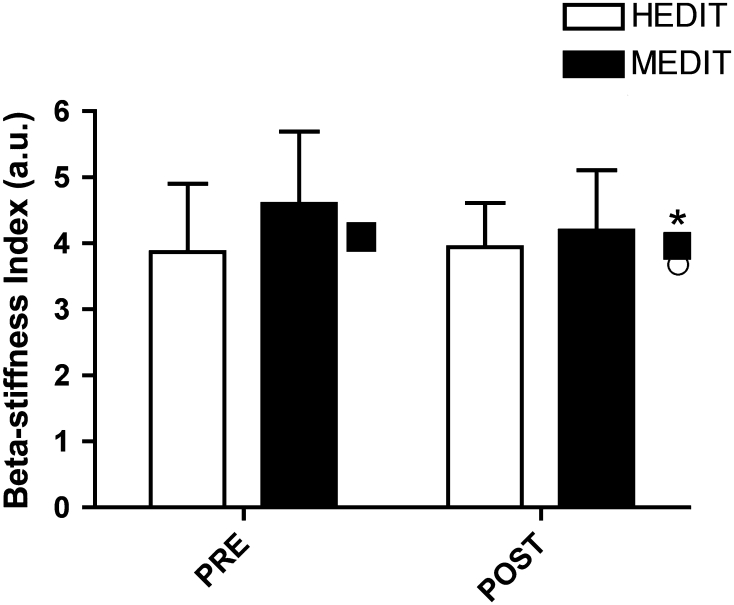
Carotid artery β-stiffness index was decreased with training (main effect for time: *p* < 0.01) as illustrated by adjusted means (HEDIT: *open circle*, MEDIT: *filled square*).*Significant adjusted change with training

Carotid minimum and maximum diameters were unaltered in either group. There were differences at PRE in cross-sectional area change (HEDIT PRE: 6.5 ± 1.4 mm^2^ vs. MEDIT PRE: 5.0 ± 1.2 mm^2^, *p* = 0.03). Subsequent ANCOVA analysis showed no differences with training (*p* = 0.53). β-stiffness index was also different at PRE (HEDIT PRE: 3.9 ± 1.0 a.u. vs. MEDIT PRE: 4.6 ± 1.1 a.u., *p* = 0.05) and ANCOVA analysis indicated a significant decrease with training (time effect: *p* < 0.01, Fig. 2), but no interaction (*p* = 0.73). Carotid pressure waveform analysis showed no changes in reflection time and AIx with training (Table 1). Carotid IMT and peripheral PWV were also unchanged (Table 1).

### Heart rate dynamics at rest

Table 2 summarizes heart rate dynamics variables. R–R interval increased with training by 44 ms (*p* = 0.05), but no interaction was apparent (*p* = 0.14). SDNN was different at PRE between groups (HEDIT PRE: 56.2 ± 5.4 ms vs. MEDIT PRE: 39.9 ± 6.0 ms, *p* = 0.03) and ANCOVA results showed no change with training (Table 2, Time effect *p* = 0.12), nor an interaction (*p* = 0.90). Adjustment at PRE (HEDIT PRE: 64.9 ± 7.5 ms vs. MEDIT PRE: 40.8 ± 8.2 ms, *p* = 0.02) and a trend towards an increase with training was noted with RMSSD (Table 2, time effect *p* = 0.06). The triangulation index (HEDIT PRE: 12.2 ± 4.1 a.u. vs. MEDIT PRE: 8.5 ± 2.2 a.u., *p* = 0.03), analysed by ANCOVA, was not altered with training (Table 2, Time effect *p* = 0.33), and there was no interaction (*p* = 0.16).

**Table 2:** Heart rate variability and non-linear dynamics before (PRE) and after (POST) 6 weeks of moderate intensity interval (MEDIT) or heavy intensity interval training (HEDIT)

	Moderate (*n* = 9)		High (*n* = 11)		*p* value time
	PRE	POST	PRE	POST	
R–R interval (ms)	863 ± 116	950 ± 177	945 ± 116	958 ± 108	0.05
NN50^†^ (count)	70.3 ± 0	97.2 ± 11.7	70.3 ± 0	85.6 ± 10.5	0.13
SDNN^†^ (ms)	45.0 ± 0	53.3 ± 5.8	45.0 ± 0	52.3 ± 5.2	0.12
RMSSD^†^ (ms)	48.9 ± 0	61.8 ± 8.8	48.9 ± 0	57.2 ± 7.9	0.06
HRV triangular index	11.7 ± 3.8	13.6 ± 4.8	15.4 ± 5.1	15.8 ± 4.4	0.33
Spectral parameters					
Total power (ms^2^)^†^	2221 ± 0	2858 ± 608	2221 ± 0	3145 ± 542	0.02
LF (ms^2^)^†^	1016 ± 0	1295 ± 302	1016 ± 0	1456 ± 271	0.05
HF (ms^2^)^†^	1070 ± 0	1504 ± 321	1070 ± 0	1371 ± 360	0.10
LF (n.u)	62.1 ± 20.8*	51.3 ± 21.3*	47.0 ± 15.7	53.2 ± 16.2	0.20
HF (n.u.)	37.9 ± 20.8*	48.7 ± 21.3*	53.0 ± 15.7	46.8 ± 16.2	0.20
LF/HF^†^	1.67 ± 0*	1.02 ± 0.18*	1.67 ± 0	1.83 ± 0.20	0.05
|1 − α_1_|	−0.13 ± 0.27*	0.03 ± 0.28*	0.07 ± 0.21^#^	−0.02 ± 0.23	0.42^#^
SD_1_	23.2 ± 10.1	35.0 ± 20.5	44.4 ± 21.2	48.2 ± 22.05	0.06
SampEn	1.50 ± 0.21	1.50 ± 0.28	1.55 ± 0.29	1.57 ± 0.14	0.80

*NN50* number of sequential N–N intervals differing by longer than 50 ms, *SDNN* standard deviation of the normalized R–R intervals, *RMSSD* root mean squared of the standard deviation, *LF* low frequency, *HF* high frequency, *1* *−* *α*_*1*_ difference from 1.0 of the detrended fluctuation analysis alpha 1 relationship, *SD*_*1*_ geometric parameter of the Poincaré plot, *SampEn* sample entropy

* Significant time × group interaction (*p* < 0.05) with means differing from each other

^*#*^ *p* values of time effects correspond to ANOVA when there were no differences at PRE and ANCOVA analysis when differences at PRE occurred (see “Results”)

^†^Adjusted means from the ANCOVA are presented

Total (HEDIT PRE: 3,038 ± 2071 ms^2^ vs. MEDIT PRE: 1,221 ± 646 ms^2^, *p* < 0.01), LF (HEDIT PRE: 1,308 ± 914 ms^2^ vs. MEDIT PRE: 657 ± 359 ms^2^, *p* = 0.02) and HF (HEDIT PRE: 1,549 ± 1243 ms^2^ vs. MEDIT PRE: 484 ± 405 ms^2^ *p* = 0.02) absolute powers differed at PRE between groups and ANCOVA analyses showed that total absolute power and absolute LF power increased by ~35 % with training (Table 2, time effects *p* = 0.02 and 0.05, respectively) with no group interaction (Table 2, *p* > 0.05). There was a trend for increased absolute HF power with training (*p* = 0.10) that may relate to inadequate statistical power. There was no interaction (Table 2, *p* = 0.80).

Normalized LF and HF showed a significant interaction with a 10.8 % decrease in normalized LF in the MEDIT group (Table 2, pairwise comparison *p* = 0.05), but no change in the HEDIT group following training (Table 2, pairwise comparison *p* = 0.31). Normalized HF showed an inverse response (Table 2, pairwise comparison *p* = 0.05). LF/HF differed at PRE (HEDIT PRE: 2.4 ± 1.8 vs. MEDIT PRE: 1.1 ± 0.7, *p* = 0.03) and ANCOVA analysis indicated a significant interaction (*p* < 0.05, Table 2). The MEDIT group displayed a ~64 % decrease (Table 2, pairwise comparison *p* < 0.01), while the HEDIT group showed no change following training (Table 2, pairwise comparison *p* = 0.40).

$| { 1.0 \, - \alpha_{ 1} } |$ showed a significant interaction with values closer to 1.0 with training; however, at PRE the HEDIT group displayed values above 1.0 while the MEDIT had values below 1.0 (Table 2). Sample entropy was not altered with training in either group (*p* = 0.80, Table 2). SD_1_ showed a trend for increase but did not reach statistical significance (*p* = 0.06, Table 2) and no interaction was observed (*p* = 0.31).

### Linear regression analysis of arterial stiffness

**Fig. 3:**
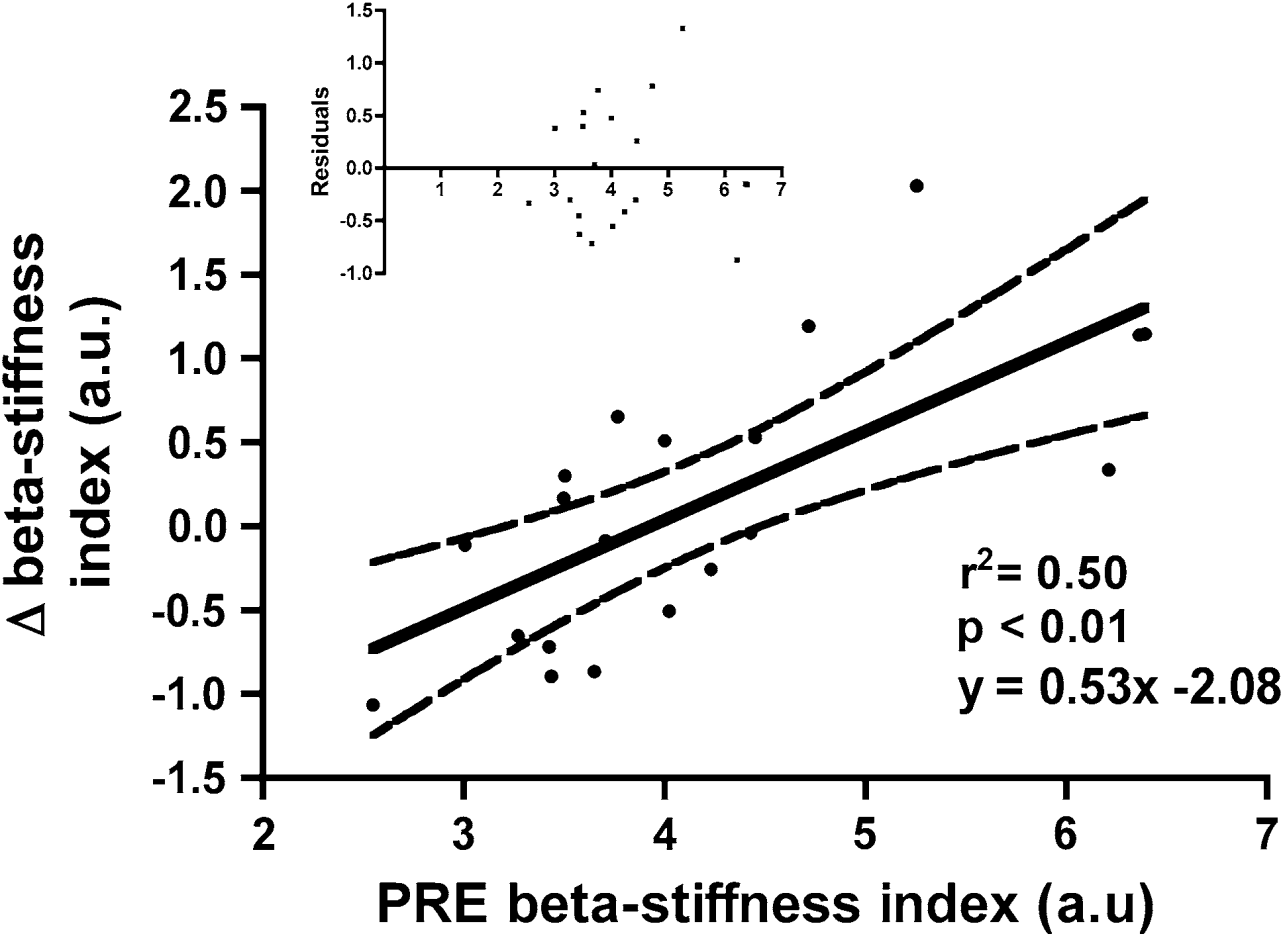
Arterial stiffness and training-induced adaptations. Linear regression analysis of β-stiffness index at PRE and the change of β-stiffness index with training. There was a significant relationship (*r* ^*2*^ = 0.50, *p* < 0.01) indicating those with highest stiffness at PRE showed the greatest reductions. Residuals are displayed in the inlay graph with random distribution indicating a good linear fit of the relationship

Linear regression analysis of the relationship between carotid artery β-stiffness index at PRE and changes in carotid artery β-stiffness index with training revealed a significant linear relationship (*r* ^*2*^ = 0.50, *p* < 0.01, Fig. 3). However, $\dot{V}{\text{O}}_{{ 2 {\text{peak}}}}$ and arterial stiffness at PRE (*r* = −0.26, *p* = 0.26), and their changes with training (*r* = −0.04, *p* = 0.87) were unrelated. Finally, changes in cardiac autonomic modulations represented by changes of the LF/HF ratio (*r* = 0.14, *p* = 0.57) or |1 − α_1_| (*r* = −0.23, *p* = 0.34) did not relate to arterial stiffness changes.

## Discussion

This is the first study to show that interval exercise training effectively alters carotid artery arterial stiffness and that the metabolic stress of the training sessions is not an important modulator. In addition, enhanced vagal activity and preferable cardiac autonomic modulations are achieved by interval exercise training but these changes were independent of improvements in carotid artery stiffness. Furthermore, improved carotid arterial stiffness was independent of improvements in $\dot{V}{\text{O}}_{{ 2 {\text{peak}}}}$, whilst higher PRE arterial stiffness appeared to be a prerequisite for change.

### Arterial stiffness improvements with interval training

Endurance exercise training can effectively improve central arterial stiffness while sprint interval training improves peripheral stiffness in the arteries of the exercised trained limbs (Cameron and Dart 1994; Tanaka et al. 2000; Rakobowchuk et al. 2008). The current study demonstrates for the first time reduced central stiffness with interval exercise training. These adaptations were independent of the metabolic stress of the training, which is somewhat in contrast to longitudinal data (van de Laar et al. 2010). These apparent discrepancies may relate to differing definitions of exercise intensity between studies. Vigorous activity defined by epidemiologists may include exercise either side of the lactate threshold. Certainly, for vascular adaptation intermittent exercise is an effective method to alter arterial stiffness, even when the metabolic stress is modest, and this should be emphasised. As such a wide variety of activities may be prescribed to ameliorate arterial stiffness and potentially improve baroreceptor function in some populations.

Our results suggest that the main modulator of improved arterial stiffness with interval training is elevated PRE arterial stiffness. Previous work in young healthy participants indicated no improvements in central arterial stiffness following either sprint interval training or endurance training (Rakobowchuk et al. 2008). However, an improvement in carotid artery stiffness in this study suggests that in the young general population there is variability in arterial stiffening and non-pharmacological treatment involving lifestyle interventions should be promoted.

Measures of peripheral arterial stiffness (PWV of the upper limb) were not altered with interval training. This is a common finding with endurance training (Petersen et al. 2006; Cook et al. 2006) and our work extends this to several exercise domains of interval training programmes. These results also suggest the alterations of central stiffness with training were not due to systemic factors such as reduced sympathetic nervous activity, systemic alterations of antioxidant status or circulating vasoactive substances. Rather altered structural elements, possibly collagen and elastin composition or reduced cross-linking may be involved (Maeda et al., 2005). These mechanisms are also supported by the lack of changes in the morphology of the carotid pressure wave (AIx and reflection time) and no alteration of left ventricular ejection duration, which may have confounded the measurements of β-stiffness if they had altered with training. The lack of change of IMT, and carotid artery dimensions further support tissue reorganisation rather than simple enlargement.

### Heart rate dynamics with interval training of differing metabolic stress

Similar to alterations of carotid arterial stiffness, heart rate dynamics were favourably altered with training, which supports previous work (Seals and Chase 1989; Goldsmith et al. 1992; al-Ani et al. 1996; Tulppo et al. 2003; Sloan et al. 2009). We show a trend for increased absolute HF power, increased absolute LF power, and an increased overall spectral power in both groups. When normalised to total power, the MEDIT group had reduced normalised LF and increased normalised HF power, reducing the LF/HF ratio; however, this altered ratio was not apparent amongst HEDIT participants. Regarding |1 − α_1_|, both groups’ values were reduced suggesting an improvement in non-linear heart rate dynamics and these changes were mirrored by training-related increases in overall HRV as supported by trends for increased SD_1_ and RMSSD. In relation to previous work evaluating dose–response relationships between HRV and training quantified by the training impulse method (TRIMP) (Iwasaki et al. 2003; Okazaki et al. 2005; Manzi et al. 2009), our results suggest that the MEDIT and HEDIT training programmes, although differing in metabolic stress, likely remained within a training impulse that would induce beneficial changes in HRV parameters (estimated Monthly TRIMP of ~300). According to regression modelling of the HF and LF response to training of this TRIMP magnitude, increases would be expected in both parameters and this was most evident in the HEDIT group who experienced the higher estimated TRIMP amongst our training groups.

Higher absolute HF power suggests increased parasympathetic modulation of heart rate dynamics while increased absolute LF power has been proposed to reflect changes in sympatho-vagal balance following moderate and high intensity endurance training (Tulppo et al. 2003). Alternatively, Goldstein and colleagues (Goldstein et al. 2011; Rahman et al. 2011) suggest LF power reflects baroreflex function and cardiac autonomic modulation by these sensory neurones (Rahman et al. 2011). Young apparently healthy (Iwasaki et al. 2003; Komine et al. 2009) and older participants (Monahan et al. 2001; Okazaki et al. 2005) display improved baroreflex function with training and our observation of increased absolute LF power complements this work. Whether a greater dose of interval exercise that induces high metabolic stress eventually causes an unfavourable regulation of HRV and baroreflex as noted in athletes (Iellamo et al. 2002) requires detailed study.

The discordant alterations in normalized LF and HF power and the LF/HF ratio between the MEDIT and HEDIT groups result from a small increase in normalized LF compared to the large normalized HF amongst the MEDIT group, while the HEDIT showed similar increases in both parameters. Traditionally these results would be interpreted as improved sympatho-vagal balance (decreased LF/HF ratio) amongst the MEDIT group (Goldstein et al. 2011), but could also suggest improved baroreflex function combined with enhanced vagal-mediated cardiac autonomic modulation in both groups (Goldstein et al. 2011), although the magnitude of these changes may differ. Whether these changes are a result of differing autonomic adaptations within interval training exercise programmes involving vastly different metabolic stress is not clear. Mechanistically, differences in the hemodynamic oscillations experienced during the exercise sessions may be involved or alterations of intrinsic heart rate, S-A node sensitivity (Bolter et al. 1973) and/or alterations of myocardial phenotype (Barbier et al. 2006).

### $\dot{V}{\text{O}}_{{ 2 {\text{peak}}}}$ improvements with high-intensity and disassociation of $\dot{V}{\text{O}}_{{ 2 {\text{peak}}}}$ improvements from arterial stiffness changes

$\dot{V}{\text{O}}_{{ 2 {\text{peak}}}}$ and arterial stiffness changes were not associated. $\dot{V}{\text{O}}_{{ 2 {\text{peak}}}}$ improvements only occurred in the HEDIT group. The HEDIT group exercise sessions likely induced mitochondrial biogenesis while the MEDIT duty cycle induced a vascular stimulus, but had little impact on mitochondrial proliferation. This supports the argument that high intensity exercise effectively improves maximal aerobic capacity and thus may be more ‘time-efficient’ in this regard (Wisløff et al. 2007; Gibala and McGee 2008). Conversely, this suggests a disassociation between metabolic stress and vascular adaptations.

## Limitations of the study

Limitations of the study may affect interpretation of the findings. Stroke volume changes can impact carotid β-stiffness index (Myers et al. 2002). However, left ventricular ejection duration and the systolic pressure area were not altered in either group suggesting no alterations in stroke volume. HRV measures were obtained during spontaneous breathing in order to maintain a physiologically relevant situation. Despite this potentially less sensitive method of cardiac autonomic function evaluation, we observed training-related alterations. This adds to the relevance of these findings since they were not simply observed during constrained paced breathing, which improves the translation of our results to normal behaviours like sleep and rest. In addition, spontaneous breathing methods are used in similar training studies by other research groups (Iwasaki et al. 2003; Manzi et al. 2009). Finally, we did not confirm whether the training stimulus of individuals was precisely above or below the LT, however, several lines of reasoning suggests this was likely true or at the very least a large difference in metabolic stress was evident between our two training programmes. First, we conducted our testing protocols virtually identically to those of Turner et al. (2006), who specifically used a ramp incremental exercise testing to determine peak work rates and training intensities. In their protocol, they explicitly show little to no lactate accumulation with exercise involving 10 s:20 s (work:rest) duty cycles while blood lactates of ~5 mmol were apparent when 30 s:60 s (work:rest) duty cycles were employed supporting vastly different metabolic stresses. Secondly, our participants were of a similar relative aerobic fitness and had similar relative _e_LT (53 %) compared to those of Turner et al. (2006) (~55 %) further supporting the likelihood that acute responses to exercise would have been similar to those outlined by Turner et al. (2006). Finally, although difficult and anecdotal evidence supporting participants fatigue, the HEDIT group were able to complete the full 30 min protocol, suggesting the overall stimulus was below the CP since exercise above this work rate leads to fatigue and exercise intolerance within 30 min of exercise initiation.

## Perspectives and significance

Similar to previous work examining exercise training as a method to improve autonomic function, our findings illustrate that even within the moderate exercise domain, benefits may be gained from interval training. Therefore, in relation to arterial stiffness and autonomic function, it is not likely that metabolic stress modulates training-induced alterations, but rather the frequent alterations of BP and blood flow likely contribute most to these adaptations, although this may differ depending on the level of the arterial system studied (Laughlin et al. 2004). In addition, a significant modulator of improved arterial stiffness in apparently healthy young adults is the initial stiffness of the arteries prior to training, which is impaired in some individuals at an early age.
